# First Results of a New Vacuum Plasma Sprayed (VPS) Titanium-Coated Carbon/PEEK Composite Cage for Lumbar Interbody Fusion

**DOI:** 10.3390/jfb9010023

**Published:** 2018-03-14

**Authors:** Sven Hoppe, Christoph E. Albers, Tarek Elfiky, Moritz C. Deml, Helena Milavec, Sebastian F. Bigdon, Lorin M. Benneker

**Affiliations:** 1 Department of Orthopedic Surgery and Traumatology, Spine Unit, Inselspital, Bern University Hospital, Bern 3010, Switzerland; christoph.albers@insel.ch (C.E.A.); moritz.deml@insel.ch (M.C.D.); helena.milavec@insel.ch (H.M.); sebastian.bigdon@insel.ch (S.F.B.); lorin.benneker@insel.ch (L.M.B.); 2 Spine Surgery Unit, El-Hadra University Hospital, Alexandria University, Alexandria 21561, Egypt; tarekfiky@yahoo.com

**Keywords:** spine, cage, titanium-coated carbon, PEEK, composite, lumbar fusion

## Abstract

The aim of this study was to assess the performance of a new vacuum plasma sprayed (VPS) titanium-coated carbon/polyetheretherketone (PEEK) cage under first use clinical conditions. Forty-two patients who underwent a one or two segment transforaminal lumbar interbody fusion (TLIF) procedure with a new Ca/PEEK composite cage between 2012 and 2016 were retrospectively identified by an electronic patient chart review. Fusion rates (using X-ray), patient’s satisfaction, and complications were followed up for two years. A total of 90.4% of the patients were pain-free and satisfied after a follow up (FU) period of 29.1 ± 9 (range 24–39) months. A mean increase of 3° in segmental lordosis in the early period (*p* = 0.002) returned to preoperative levels at final follow-ups. According to the Bridwell classification, the mean 24-month G1 fusion rate was calculated as 93.6% and the G2 as 6.4%. No radiolucency around the cage (G3) or clear pseudarthrosis could be seen (G4). In conclusion, biological properties of the inert, hydrophobic surface, which is the main disadvantage of PEEK, can be improved with VPS titanium coating, so that the carbon/PEEK composite cage, which has great advantages in respect of biomechanical properties, can be used safely in TLIF surgery. High fusion rates, good clinical outcome, and low implant-related complication rates without the need to use rhBMP or additional iliac bone graft can be achieved.

## 1. Introduction

Lumbar interbody fusion with decompression is a standard method used in the treatment of degenerative spinal diseases that result in spinal stenosis, spondylolisthesis, or segmental instability. Its aim is the elimination of mechanical pressure on neural elements (spinal cord, spinal nerves) by direct and indirect decompression, restoration of disc height, and establishing primary stabilization of the spine with an appropriate alignment.

Cage design, mechanical properties, surface topography, and material influence load bearing capacity, primary stability, alignment, osteointegration and fusion, as well as handling properties. The geometric design of the cage used for fusion is an important factor for enabling physiologic spinal alignment. Wedge-shaped cages are successful in the correction of physiologic lumbar sagittal alignment and in restoring intervertebral height [1]. Standard cages are usually made from titanium or similar alloys with enough mechanical strength to withstand the high load bearing and present good handling properties, primary stability, and biocompatibility that facilitate rapid osteointegration. One disadvantage of metallic implants is the mismatch in stiffness as compared to the surrounding bone, which can lead to subsidence of the cage into the endplates. The Young’s modulus of titanium alloys is 110 GPa, much higher as compared to cancellous or cortical bone (respectively 2, 18 GPa); polyetheretherketone (PEEK) cages present a stiffness close to that of trabecular bone (3–4 GPa) whereas the stiffness of carbon fiber-reinforced PEEK composite material can be tailored to match the modulus of cortical bone by varying the fiber content and orientation [2,3,4].

Both PEEK and carbon fiber/PEEK (Ca/PEEK) composite cages are radiolucent and require markers to control positioning of the cage during surgery. The advantage compared to metallic implants is the artifact-free compatibility for computer tomography (CT) and magnet resonance imaging (MRI) examinations, which can be helpful to evaluate interbody fusion, neurocompression, or other intraspinal pathologies.

A clear disadvantage of PEEK-based implants is their smooth and hydrophobic surface resulting in a poorer primary stability; the resulting low surface energy has a negative effect on bone growth onto the implant, which is why many surgeons use expensive growth factors, such as recombinant bone morphogenetic protein (rhBMP), to improve interbody fusion [5,6]. One strategy to improve primary stability and osteointegration is to modify the implant’s surface by chemical or physical treatment or by adding an osteoconductive layer, usually hydroxyapatite or titanium, and thereby increase the contact area and roughness [7]. The ideal roughness of implant surfaces is considered in the micrometer-scale between 1.5–80 micrometers [8]. In Ca/PEEK implants any physical or chemical treatment would expose the carbon fibers, therefore, low-temperature spraying techniques have to be used to create an osteoconductive coating on the implant.

Experience with coating medical/orthopedic implants with titanium reach back over 30 years. In vitro and in vivo studies showed that titanium- and titanium alloy-coated implants promote osteointegration by stimulation of osteoblasts and the reduction of osteoclast activity [9,10]. Vacuum plasma sprayed (VPS) coating provides a roughness of 6 to 12 micrometers. With VPS titanium-coated screws showed improved bone apposition resulting in greater removal torque compared to uncoated and with physical vapor deposition processed screws [6]. The porous titanium-coated surface may provide an increased fusion interface between the end plate and the cage [11].

With the use of a VPS-coated cage it was, therefore, aimed to increase the osteoconductive effect with the important advantages of the obtained selective roughness and porosity, although some studies have shown that a rough implant surface increases primary implant stability and accelerates colonization of the blood cells and development of new bone tissue [8,9].

The aim of this study was to assess the performance of a new vacuum plasma sprayed (VPS) titanium-coated carbon/PEEK cage (E-Turn cage^®^, Icotec AG, Altstätten, Switzerland) under first use clinical conditions with focus on fusion rates, patient’s satisfaction, and complications.

## 2. Results

The mean time of surgery was 160 ± 54 (standard deviation, SD) (range 80–300) min. The mean blood loss was 564 ± 455 (range 150–2000) mL. The mean time of hospitalization was 6 ± 2 (range 3–14) days. The FU period was 29.1 ± 9 (range 24–39) months. According to the clinical evaluation, 90.4% of the patients were pain-free and satisfied. Preoperatively, 9.6% of patients had the same symptoms.

Radiologically, the rate of G3 degeneration in the adjacent segment discs increased significantly by 11% between preoperative and final follow-up radiographs.

Although there was a mean increase of 3° in the segmental lordosis in the early period (*p* < 0.05), it was observed to have returned back to preoperative levels at final follow-ups (Table 1). No change was observed in the angle of adjacent segment lordosis or global lumbar lordosis between preoperative and the last follow-ups. In two patients, a pedicle screw loosening angle of 2° and/or above was observed, but these patients were asymptomatic and the cage was fully integrated.

In the final follow-up, the segmental height Mochida index and foraminal height Mochida index were 5.8 and 3.82, respectively, as compared to the early postoperative radiograph. An increase in segmental and foraminal height Mochida index in between preoperative and final follow-up were 6.89 and 5.56, respectively.

According to the Bridwell classification, the mean 24-month G1 fusion rate was calculated as 93.6% and the G2 as 6.4%, and no cases with radiolucency around the cage (G3) or clear pseudarthrosis could be seen (G4). Table 2 lists complications in the short-term follow-up. One patient underwent an operation due to adjacent segment disease

## 3. Discussion

The aim of this study was to evaluate the reliability and effectiveness of Ca/PEEK cages produced with the VPS titanium-coating method to increase the bone ongrowth and osteoconductive properties, which can be used to apply transforaminal lumbar fusion (TLIF) and posterior lumbar interbody fusion (PLIF) for degenerative spinal disease.

The most important result of this study was that in the vast majority (90.4%) of patients, a significant degree of clinical improvement was achieved in addition to radiological union in all patients. Appropriate disc height and restoration of segmental lordosis in single and two-level lumbar fusion are of great importance in respect of short- and long-term results. By increasing segmental and foraminal height, as represented by values on the Mochida Index of 6.89 and 5.56, respectively, on the final follow-up radiographs compared to pre-operative values, the space for the spinal nerves in the foramen and the disc spaces were significantly restored.

Ca/PEEK is an ideal intervertebral fusion material as it is physically and chemically stable with an elastic modulus similar to cortical bone and the bioactive properties of the biologically inert surface can be increased. Two basic strategies have been developed to increase the bioactive properties of the Ca/PEEK surface. The first option is the adsorption of bioactive materials, such as hydroxyapatite, bioglass, calcium silicate, or glass-ceramic on PEEK cages [12]. Despite the potential advantages of rapid osteointegration of the bioactive treatment and no requirement for allograft or autograft, it is not a readily available material for clinical application because of the difficulty of production, cost, the change in physical properties, and that it is still in the development stage [13]. The second strategy is surface modification. Hydroxyapatite and titanium are the most commonly used materials for surface modification. Titanium is an ideal material for coating the Ca/PEEK surface because, in the surface coating process, the PEEK substrate is not negatively affected as crystallization is achieved easily without any need for heat treatment in the titanium deposition process [5]. A previous study reported Ca/PEEK screws coated with titanium using the 2 different methods of VPS or physical vapor deposition [6]. The titanium-coated Ca/PEEK screws were implanted in sheep tibia and compared to a control group with uncoated Ca/PEEK screws. There was a significantly increased bone deposition in group with Ca/PEEK screws. However, the torque required to remove the VPS-coated titanium screws was statistically significantly greater compared to the uncoated screws or the PVD-coated titanium screws. This has been shown to increase the bone ongrowth quality. According to a study by Borsari et al., VPS technology offers significant advantages for depositing oxygen-sensitive materials [14], some of which are that the roughness and porosity of the implant surface can be selected, a well-bonded coating can be achieved together with the optimum structure and surface morphology, and long-term tight and stable fixation can develop between the implant and the tissue [14,15,16].

Comparable results for fusion and complication rates in this study could be found in the literature. Lee et al. used a PEEK cage with a local bone graft and demineralized bone graft in TLIF patients and reported 98% fusion in a 24-month follow-up period [17]. In a study by Wang et al., a PEEK cage with local bone graft and demineralized bone graft was used on TLIF patients and, at mean 26-month follow-up, rates were reported of 97% fusion [18]. In a meta-analysis by Wu et al., at a mean 26-month follow-up of TLIF patients, fusion rates of 90.9% were found [19]. According to a systemic review by Galimberti et al., the fusion rate in TLIF/PLIF patients at mean 24-month follow-up was 89.5% and, in PLIF/TLIF patients in which rhBMP-2 was used, the fusion rate was 95.7% [20].

Lumbar fusion made with the interbody fusion cage in combination with autograft taken from the iliac wing is the standard method. However, donor site morbidity is still a significant problem. Therefore, in recent studies, some alternative methods to autograft taken from the iliac wing have been reported. In a study by Villavicencio et al., PLIF was applied to patients using rhBMP and local graft with a PEEK cage and at 10 months follow-up, 100% union was achieved [21]. In another study by Mummaneni et al., PEEK cage with rhBMP and local autograft was applied to 21 patients and union was achieved in 20 of the 21 patients [22]. However, despite the high fusion rates with the use of rhBMP, significant problems have also been reported. In a study by Singh et al., minimally invasive TLIF with rhBMP-2 was applied to 587 patients [23]. Postoperative radiculitis developed in 57% of patients and complications which required revision occurred in 49 patients (9.3%), because of pseudarthrosis in 39 and symptomatic neuroforaminal bone formation and/or cage migration in 10 patients. Vaidya et al. reported that revision was required because of cage migration that led to neurological problems in 33% of TLIF cases [24]. Ectopic bone formation of rhBMP, especially in the neuroforaminal region is an important problem. Apart from these complications, high costs are another important point. The significant complications and morbidity reported in studies made with PEEK led us to the modification of the PEEK cage. 

Several studies have focused on osteointegration increased through the manipulation of the surface topography, which is a major factor for osteointegration [25]. It was aimed to increase the osteoconductive effect with the important advantages of selective roughness and porosity obtained with the VPS method. The results of this study support that this method makes a positive contribution to clinical results. In addition, some studies have shown that a rough implant surface increases the primary stability of the implant, and accelerates colonization of the blood cells and the development of new bone tissue [8,9].

Subsidence following TLIF and PLIF surgery is a significant problem seen particularly in elderly patients with low bone mineral density. The advantage of cages with large footprint design is that the cage-bone fusion area is widened. Another important advantage is to reduce the load on each area, micro-fractures in the vertebra end plates and disc area collapse may be prevented. Biomechanical experimental studies have supported this view [26]. 

There are some limitations to this study. First, the fusion was evaluated only by plain radiographs. In the literature, radiological evaluation of fusion is often made with both CT and plain radiographs. As only few of our patients received CT scans we did not include this data. Second, the design of the study is retrospective and there has not been a control group with interbody fusion using Ca/PEEK cages uncoated or coated with other materials. Nevertheless, as this study only aims for first results this might be of minor interest.

## 4. Materials and Methods

All patients who underwent a one- or two-segment TLIF procedure with a new Ca/PEEK composite cage between 2012 and 2016 were retrospectively identified by an electronic patient chart review (86 patients). The study was approved by the local ethic committee and informed consent was signed by all participants.

Indications for surgery were spinal stenosis, degenerative spondylolisthesis, isthmic spondylolisthesis, and recurrent degenerative disc disease. Patients’ demographic information are given in Table 3. Pedicle screws were used in all cases for single or multiple levels in the anterior wedge interbody fusion cage. Multiple level fusions were performed in five patients (11.9%).

Preoperative, early postoperative (six months postoperative), and at final follow-up (two years postoperative) radiological assessments (X-ray), as well as clinical information of the patients, were obtained.

### 4.1. Inclusion/Exclusion Criteria

Patients with incomplete data (27 patients), active malignancy (four patients), trauma (four patients), or previous lumbar spine fusion surgery (nine patients) were excluded. This left 42 patients for analysis.

### 4.2. Implant

**Figure 1:**
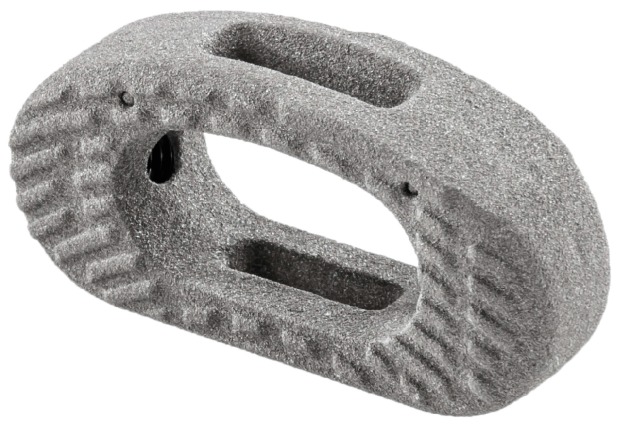
E-turn cage.

The E-turn (E-Turn cage^®^, Icotec, Switzerland) (Figure 1) is an elliptically-formed Ca/PEEK cage with a 5° slope with a large central chamber for the apposition of autologous or allogenic bone, demineralized bone matrix, or osteoinductive material. Ca/PEEK composites are radiolucent thus reducing artifacts in post-operative imaging [27]. To ensure correct positioning intra-operatively using the C-arm, four tantulum markers are integrated in the four ventral corners of the cage. A 70 micrometer thick Ti-coating is applied using the VPS coating technique [28], which provides a biocompatible interface and optimal roughness. Slightly curved grooves on both the cranial and caudal surface should facilitate intra-operative positioning [27]. Compared to most other cage designs, a larger footprint further increases the bone-cage interface, thus providing increased stability and supporting osteointegration [26]. The E-turn cage is suitable for TLIF and PLIF providing self-distraction and, thus, correct anatomical height and sagittal alignment.

### 4.3. Surgical Technique

All cages were inserted in a TLIF technique from the posterior of the site of greater compression or symptoms [5] by three senior orthopedic spine surgeons at one institution. A midline incision was used for open decompression, clearance of the disc space and insertion of the cage. All patients received an additional posterior stabilization with pedicle screw/rod constructs (USSII, Synthes Bettlach, Switzerland). Cage and remaining disc space were filled with autologous bone harvested from the decompression or facet joint resection.

### 4.4. Clinical Evaluation

Postoperative subjective outcome of surgery was graded using a simple four-dimensional classification comparing pre-operative symptoms to symptoms at last follow-up: (1) worsening of symptoms; (2) same symptoms/no change; (3) fewer symptoms, but not pain free; and (4) pain-free and satisfied with surgical result. Operation time and blood loss was obtained from anesthesiology log files. For evaluation of postoperative complications and length of hospital stay a complete electronic patient chart review was performed searching for compromised cicatriation, thromboembolism, surgical site infection, peripheral neurological deficit, and implant failure.

### 4.5. Radiological Evaluation

**Figure 2:**
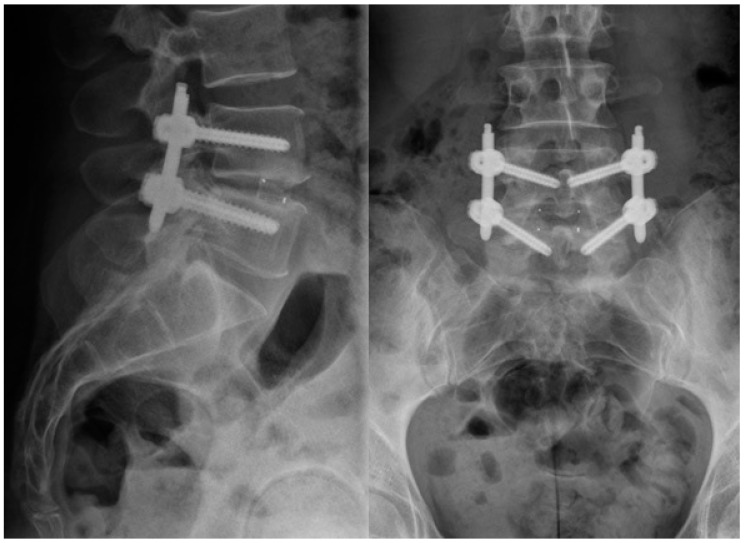
anterior–posterior (**right**) and lateral (**left**) X-ray of an 40 year old woman after TLIF surgery with percutaneous dorsal instrumentation and implantation of the newly-developed cage at the level L4/5, one-year FU.

The radiologic assessment was performed in consensus by an independent, board-certified, experienced spine surgeon and an independent radiologist, which were not involved in the surgeries or patient’s aftercare. For the radiologic evaluation, preoperative and early postoperative (six months) antero-posterior (AP) and lateral plane standing radiographs (Figure 2) were used. Lumbar lordosis, as well as segmental lordosis of the operated and the adjacent levels, were measured at all time points using Sectra Workstation IDS7 (Version 16.2.4.2112, Sectra AG, Linköping, Sweden, 2014). Radiologic fusion was evaluated according to the interbody fusion grading system of Bridwell in the latest available radiograph [29] (Table 4).

In order to evaluate the early degeneration in adjacent discs, a validated radiological classification system, which is based on height loss in disc, type and size of osteophytes, Schmorl’s nodes, intradiscal calcification, sclerosis, and endplate shape was used (Table 5) [30]. Disc height was measured according to Frobin et al. [31].

**Figure 3:**
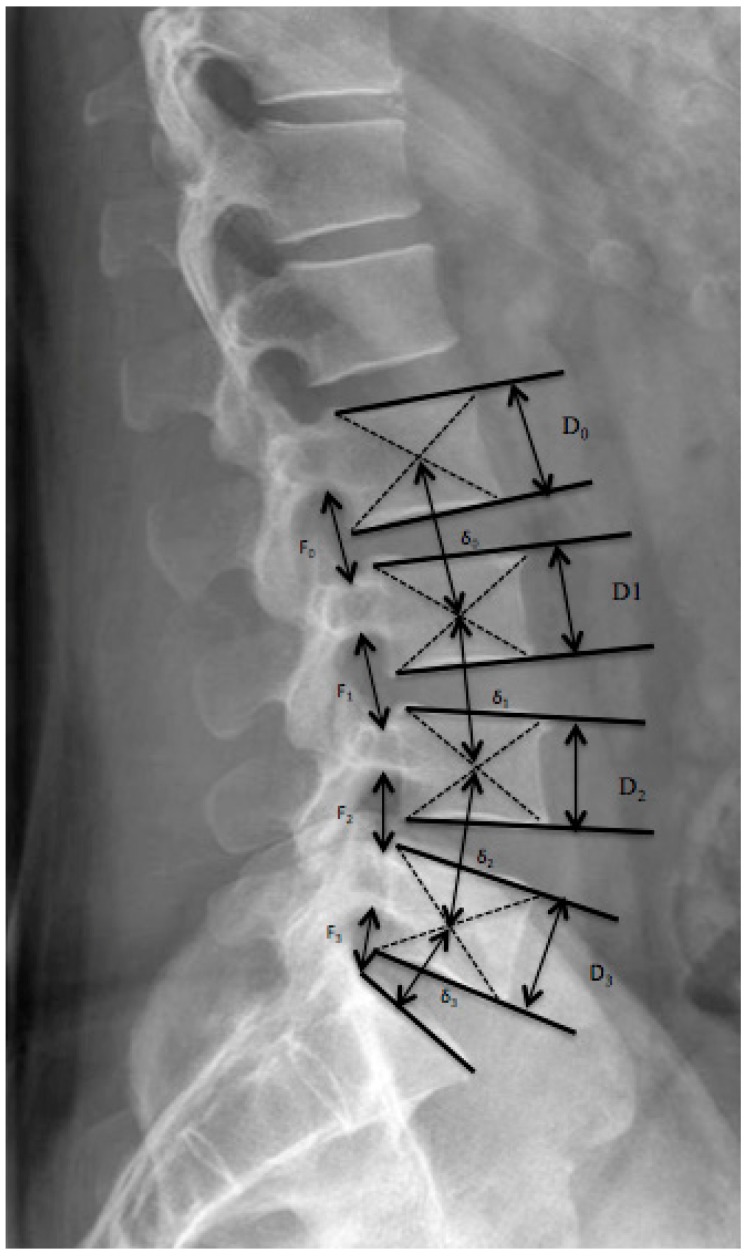
Normalized foraminal height was evaluated by dividing the measured foraminal height F through the height of the vertebral body D just beyond, according to the formula Fnorm = F/D. Measurement of the normalized segmental height Snorm = δ/D.

Since disc limits cannot be clearly defined due to intervertebral fusion within the follow-up periods, segmental height was measured instead of disc height. Normalized foraminal height was evaluated (Figure 3). For the segmental height and loss of disc height observed over time, the Mochida index was used after modification to eliminate the real size of patient and radiologic magnification [32,33].

In order to evaluate possible loosening of pedicle screws, the angle between the bisector of the axes of the two pedicle screws at the top of the instrumentation level, and the upper end plate was compared in the early postoperative and last follow-up radiographs. A change of two degrees or more was considered as a loosening of the screws [3]

All statistical analysis has been performed using IBM SPSS Statistics 21.0 (IBM Corp. Released 2012. IBM SPSS Statistics for Windows, Version 21.0.) Pre- and postoperative values were compared using paired Student’s *t*-test for continuous and normally distributed values. Wilcoxon test was used to compare ordinal data.

## 5. Conclusions

Biological properties of the inert, hydrophobic surface, which is the main disadvantage of PEEK, can be improved with VPS titanium coating, so that the carbon/PEEK composite cage, which has great advantages in respect of biomechanical properties, can be used safely in TLIF surgery. We achieved high fusion rates, good clinical outcome, and low implant-related complication rates without the need to use rhBMP or additional iliac bone graft.

## Figures and Tables

**Table 1:** Segmental, caudal adjacent, cranial adjacent, and global lordosis preoperative, six months postoperative, and at latest FU, and degeneration at adjacent segment pre-OP and at latest FU; * (*p* > 0.05).

	Pre-OP	Six Months Post-OP	Latest FU
**Lordosis**			
Segmental lordosis (range)	9° (0–14°)	12° (7–16°) *	10° (2–15°)
Caudal adjacent lordosis	10° (8–15°)	10° (7–15°)	10° (7–15°)
Cranial adjacent lordosis	11° (7–14°)	11°(7–14°)	11° (7–15°)
Lumbar lordosis L1–S1	52° (41–63°)	53° (41–63°)	53° (41–63°)
**Degeneration at adjacent segments (n = 74)**			
G0 (n)	15	14	13
G1 (n)	38	39	36
G2 (n)	15	15	14
G3 (n)	4	4	11 *

**Table 2:** Complications with respective operation level, post-operative interval until onset of symptoms, treatment, and result are listed for each patient.

Patient	Age (Years)	Operation Level	Post-Operative Interval (Days)	Complications	Treatment	Results
1	55	L4–5	5	Seroma	Clinical Observation	Resolved without intervention
2	29	L5–S1	4	Hyposensibility	Steroid	2 months later resolved
3	65	L3–4	11	Hematoma	Clinical Observation	Resolved without intervention
4	54	L3–4	3	Deep infection	5 times debridement + antibitherapy	Fully recovered
5	69	L4–5	5	L5 right partial motor deficit	Steroid	Resolved without intervention
6.	67	L4–5	4	L5 right partial motor deficit	Steroid	Resolved without intervention
7	78	L5–S1	8	Wound disorder	Clinical Observation	Resolved without intervention
8	63	L3–4	4	L5 right paraesthisia	Steroid	Resolved without intervention
9	41	L5–S1	9	L4–5 partial motor deficit + paraesthisia	Steroid	Persisting L4–5 hyposensibility + decreased force (M4)
10	63	L3–4	9	Lung embolia	Medical treatment	Fully recovered
11	28	L4–S1	4	L5 right paraesthisia	Steroid	Paraesthisia
12	62	L3–4	8	L2–3 partial motor deficit + L5 paraesthisia	Steroid	L2–3 M4

**Table 3:** Patients’ demographics.

Parameter	Value
Number of patients (levels)	42 (47)
Patients lost to follow-up (%)	27
Multiple level fusion (%)	5 (12)
Mean age (range)	59.6 (28–82)
Male (%)	23 (55)
Mean BMI (± SD, range; kg)	28.3 ± 5 (19–40)
Mean time of follow-up (± SD, range; months)	29.1 ± 9 (24–39)
Diagnosis	
Spinal stenosis (%)	26 (62)
Degenerative spondylolisthesis (%)	11 (26)
- Meyerding I (%)	7 (63)
- Meyerding II (%)	4 (37)
- Meyerding III (%)	-
- Meyerding IV (%)	-
Isthmic spondylolisthesis (%)	2 (5)
Recurrent degenerative disc disease (%)	3 (7)
Level with fusion	
L2–3 (%)	9
L3–4 (%)	17
L4–5 (%)	23
L5–S1 (%)	51

**Table 4:** Bridwell interbody fusion grading system grade description.

I	Fused with remodeling and trabeculae present
II	Graft intact, not fully remodeled and incorporated, but no lucency present
III	Graft intact, potential lucency present at top and bottom of graft
IV	Fusion absent with collapse/resorption of graft

**Table 5:** Parameters (and scores) on plain anteroposterior and lateral radiographs; disc height, osteophytes, and calcifications correlated significantly with degeneration.

Grade	Height Loss	Osteophytes	Schmorl’s Nodes	Intradiscal Calcification	Sclerosis	Endplate Shape
0	0–10%	Margins rounded	Not present	No calcifications	None	Continuous
1	10–20%	Margins pointed	Present	Rim calcification	Moderate	Irregular
2	20–30%	<2 mm	-	Intranuclear calcification	Severe	Disrupted
3	>30%	>2 mm	-	-	-	-
